# Brief Interventions in Primary Care: an Evidence Overview of Practitioner and Digital Intervention Programmes

**DOI:** 10.1007/s40429-018-0198-7

**Published:** 2018-05-03

**Authors:** Fiona Beyer, Ellen Lynch, Eileen Kaner

**Affiliations:** Institute of Health and Society, Newcastle University, Baddiley-Clark Building, Richardson Road, Newcastle upon Tyne, NE2 4AX UK

**Keywords:** Alcohol drinking, Alcohol problems, Brief interventions, Emergency care, Primary care, SBIRT

## Abstract

**Purpose of the Review:**

Excessive drinking is a major public health problem that adversely affects all parts of the population. Previous systematic reviews and meta-analyses have reported that brief interventions delivered in primary care are effective at reducing alcohol consumption, albeit with small effect sizes that have decreased over time. This review summarises the updated evidence base on practitioner and digitally delivered brief interventions.

**Recent Findings:**

Using Cochrane methodology, 69 primary care brief intervention trials (33,642 participants) and 57 digital intervention trials (34,390 participants) were identified. Meta-analyses showed both approaches significantly reduced consumption compared to controls. Five trials (390 participants) compared practitioner-delivered and digital interventions directly with no evidence of difference in outcomes at follow-up.

**Summary:**

Brief interventions have the potential to impact at both individual and population levels. Future research should focus on optimising components and delivery mechanisms, and on alcohol-related harms. Digital interventions may overcome some of the implementation barriers faced by practitioner-delivered interventions.

## Introduction

According to the World Health Organization (WHO), more than 5% of the global burden of disease and injury is attributable to alcohol consumption, and it is one of the top five risk factors for disease, disability and death throughout the world [1••]. Hazardous drinking is a pattern of drinking that increases the risk of harmful consequences [2] while harmful consumption is defined by the WHO as:

drinking which results in detrimental health and social consequences not only for the drinker, but for those around them and society at large[1••]*.*

Hazardous and harmful alcohol consumption contribute to more than 200 disease and injury conditions, most notably alcohol dependence, liver cirrhosis, suicide and trauma. Such problematic alcohol use can also exacerbate infectious diseases by weakening the immune system. The economic cost of alcohol consumption—including both health and social harms—has been estimated to total more than 1% of gross domestic product in high- and middle-income countries [3]. Although low consumption of alcohol can decrease the incidence of certain diseases and enhance feelings of well-being, these effects disappear when consumption is heavy, and the net effect of alcohol consumption is detrimental to health. Alcohol-related risk and harm are not only caused by the frequency of drinking and/or volume consumed, but by the pattern of use (e.g. high intensity single occasion or ‘binge’ drinking) and an array of contextual factors (e.g. whether alcohol is consumed with food, or car driving whilst intoxicated) [1••].

At a population level, epidemiological research shows that the majority of alcohol-related problems are not attributable to people who are dependent on or addicted to alcohol, but rather to hazardous or harmful drinkers because of the much larger size of these groups [4]. Therefore, from a public health perspective, a large impact can be made by reducing alcohol consumption in the latter groups who may well be unaware that their drinking is adversely affecting their health and well-being [5•].

## Brief Interventions in Primary Care

Use of a short screening questionnaire to identify hazardous or harmful drinking, and brief alcohol intervention in primary care is currently recommended as an evidence-based strategy to reduce alcohol consumption in people who are not seeking help for alcohol-related problems [6••]. Primary care patients are regularly asked about alcohol consumption in health checks and chronic disease management clinics, providing ample opportunities for screening. Brief intervention in the form of structured advice or condensed behaviour change counselling can also fit within routine appointment times and be delivered by generalist practitioners.

Brief interventions aim to help hazardous and/or harmful drinkers understand the risks or adverse impacts of their drinking and explore possible ways to cut down. Brief intervention is an umbrella term for advice and/or counselling that can vary in the duration or number of sessions and precise content. However, all brief interventions share similar underpinning health psychology (social cognitive and motivational) theory [7] and a component structure (commonly known as FRAMES) [8]: Feedback about existing consumption and potential/actual harms; Responsibility for change resting with the person/patient; Advice about practical strategies to reduce drinking; a Menu of options to help achieve behaviour change; Empathic delivery; and Self-efficacy building by practitioners.

Brief alcohol interventions are not considered appropriate for people who are dependent on alcohol since they require more intensive treatment with a different goal, usually abstention from alcohol rather than reduced consumption. However, brief intervention is unlikely to harm patients who may have mild to moderate dependence and the screening process can identify those needing referral for detoxification and more intensive counselling and support [9].

Despite the existence of a large evidence base on brief intervention that has growth over nearly 30 years, there is little sign that such interventions have been routinely adopted in practice [6••]. Key barriers to the delivery of brief interventions by practitioners are lack of time and low confidence about discussing alcohol with patients [10, 11]. The advent of the internet and explosion in ownership of computers and mobile devices has afforded new ways for people to access advice, information and encouragement to improve their health behaviours. As a result, brief alcohol interventions can be delivered via digital devices and platforms such as computers, websites and mobile telephone applications or ‘apps’. Digital interventions can provide a flexible, interactive and convenient way to deliver or access compared to face-to-face sessions in a health setting. Additional advantages of digital interventions include greater accessibility for potential users, regardless of geographical distance, enhanced anonymity and scope for ongoing support. For healthcare providers, digital interventions are likely to require less clinician time and less need for them to develop intervention skills via training, and potentially reduce per patient costs (especially after development) compared to direct delivery [5•]. However, some patients may not be comfortable with digital devices and may prefer conversation-based intervention.

## Evidence of Effectiveness

**Fig. 1:**
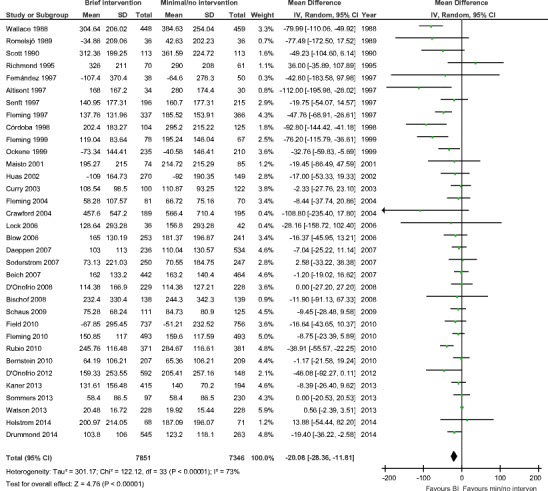
Meta-analysis of practitioner-delivered brief intervention versus minimal or no intervention. Figure is reproduced from [26••]

**Fig. 2:**
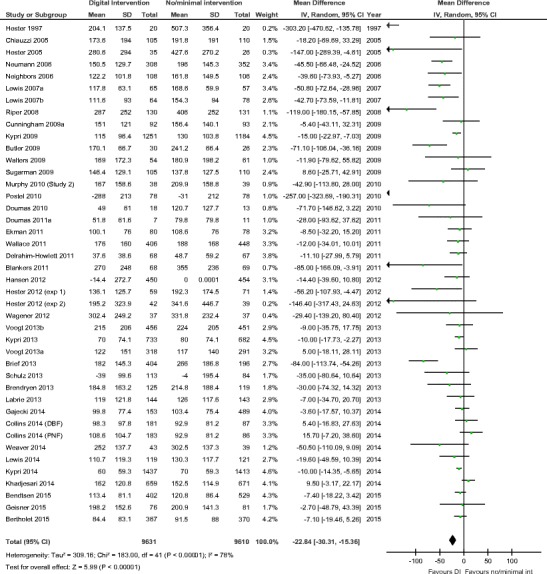
Meta-analysis of digital brief intervention versus minimal or no intervention. Figure is reproduced from [27••]

Numerous systematic reviews have assessed the effectiveness of screening and brief alcohol intervention approaches and generally conclude they are effective in reducing alcohol consumption [6••, 12•]. Some reviews have a narrower focus than others, investigating the impact of a particular type of brief intervention [13•, 14], delivery in a specific setting [15–19] or location [20, 21], or for a certain population [22–25]. This evidence base has most recently been synthesised in two linked Cochrane systematic reviews: the first was an update of a review of practitioner-delivered brief interventions in primary care settings (including emergency care) [26••]; the second focused on digital interventions to reduce hazardous and harmful alcohol consumption [27••]. The primary meta-analyses of the practitioner-focused review showed a mean reduction in consumption of 20 g per week (95% confidence interval − 12 to −28; 34 trials; Fig. 1) at 1-year follow-up. This was small decrease in mean effect size of 18 g per week since the previous review in 2007. Regarding digital alcohol interventions, these were found to reduce alcohol consumption by 23 g per week (95% confidence interval − 15 to −30; 42 trials; Fig. 2) at longest follow-up. In each case, intervention group outcomes were compared to that of controls receiving screening or assessment only, or minimal feedback plus general alcohol or health information. These recent findings equate to average reductions of about 2.5 (UK), 1.5 (USA), or 2 (EU) standard drinks. These estimates were robust to sensitivity analyses that omitted studies at higher risk of bias based on a range of methodological features of the included trials.

Although these results seem to show little difference in effect between practitioner-delivered and digital interventions, overall pooled estimates can disguise a range of effect sizes depending on participant and intervention characteristics, nature of the control condition and follow-up time points. Several subgroup analyses were carried out to unpick some of these differences. Practitioner-delivered interventions were reported to have a significant impact on consumption for both men and women in 11 trials that reported gender data separately. The effect for women was smaller but not significantly different from that of the men. Evidence on gender-specific effects was lacking for digital interventions; only five small trials reported data by gender, demonstrating no evidence of difference in effectiveness between men and women. Interventions delivered in emergency departments reduced alcohol consumption less than those delivered in general practice. However, trials in emergency settings tended to be more recent, so this finding was confounded by the fact that recent trials generally reported smaller effects. One explanation for this could be that average baseline levels of alcohol consumption in patients enrolled in recent trials tended to be considerably lower than those reported 20 or 30 years ago. Trials that recruited adolescents and younger adults reported smaller effects than those with no age restrictions for both practitioner-delivered and digital interventions. To date, only five trials (390 participants) have directly compared digital and practitioner-delivered interventions [27••], and pooling them provided no evidence that one was superior to the other. Further planned work will combine trials of digital and practitioner-delivered interventions in a network meta-analysis [28], allowing a more fine-grained comparison of different control conditions and different modalities of intervention.

## What Are the Key Components of Brief Intervention Effects?

Evidence about the most impactful features of brief alcohol interventions is beginning to emerge from the literature. The Behaviour Change Technique (BCT) Taxonomy [29] provides a systematic method for identifying individual intervention components and assessing the differential effects of their presence or absence in interventions. A secondary analysis of the Cochrane review of digital interventions [27••] assessed whether there was any association between effect size and individual BCTs [29]. Meta-regression suggested that behaviour substitution (defined as “prompt substitution of the unwanted behaviour with a wanted or neutral behaviour”) and intervention from a credible source (“present verbal or visual communication from a credible source in favour of or against the behaviour”) were associated with effectiveness [30•]. An analysis of the Cochrane review of practitioner-delivered interventions [26••], using a smaller set of alcohol-focused BCTs, suggested that interventions that encouraged self-monitoring of high risk times and situations for heavy drinking (“prompted self-recording”) were significantly associated with effectiveness [31]. These active ingredients of positive behaviour change should feature in any future development of brief intervention programmes.

The digital intervention review also examined whether reported use of theory to inform the design and development of the brief interventions had an impact on effectiveness. However, reporting of theory was too infrequent to make a robust judgement [32]. A separate meta-regression investigating a range of potential effect size modifiers of brief interventions for heavy drinking college students, including methodological characteristics, publication status, participant demographics, intervention details and outcome measurement, reported that studies using motivational interviewing, motivational enhancement therapy or personalised feedback only techniques resulted in the most consistent, positive effects [33]. A second meta-regression looked at brief interventions in emergency settings and assessed five potential effect modifiers: the type or duration of the intervention, the person delivering the intervention, the quality of the study and the nature of the control condition [34•]. They reported no evidence of impact on effect size from any of these factors.

## Heterogeneity

A feature of all meta-analyses of brief alcohol interventions, whether delivered by practitioners or via digital devices, is the high level of methodological and statistical heterogeneity. This is not surprising in a literature that has accrued over three decades. Over this period of time, views about what constitutes risk due to alcohol consumption have changed, and this is often reflected in changing national recommendations for low-risk drinking [1••]. The mean baseline consumption in trials (that reported it) of practitioner-delivered interventions published before 2007 was 313 g per week, compared to 181 g per week in trials published since then. In addition, since brief interventions are not standardised, but rather loosely based on a general framework, there can be different emphases on aspects of content in differing trials and over time. Brief alcohol interventions in primary care could also range in duration (5 to 60 min), frequency (1–5 sessions) and the practitioner delivering advice or counselling (doctors or nurses). A subgroup analysis in the Cochrane review of practitioner-delivered interventions suggested that advice-based interventions had more impact than counselling-based interventions (a reduction of 33 g of alcohol per week (95% confidence interval − 20 to − 46) versus 0.2 g per week (95% confidence interval − 3 to + 3). This finding should be treated with caution and merits further investigation, as it may be confounded by the publication date of the trials [26••].

Another element of heterogeneity in the brief intervention literature concerns the control conditions. In terms of Public Health principles of practice, there is an ethical issue regarding screening and identifying people as harmful consumers of alcohol, but then not intervening [35]. Consequently, it can be challenging to provide a ‘true’ control condition of no input. Consequently, many control conditions may contain some active ingredients of behaviour change [36]. Ideally, any activity within a control condition should not imitate elements of the intervention, but some do. Indeed, the control conditions in some brief intervention trials are longer than the active intervention conditions in other trials. This makes interpretation of the evidence in this field even more difficult, as reductions in consumption at follow-up are regularly reported in both intervention and control groups. Moreover, reporting deficiencies in trials mean that it is not always possible to tell exactly what the control group received—particularly in older trials, where the term ‘treatment as usual’ is used, typically without further explanation. Nevertheless, a subgroup analysis investigating between-group differences where controls received alcohol-related information versus those where controls received no alcohol content showed a trend towards a larger effect estimate from trials in the latter group, (−13 g per week (95% confidence interval −23 to −3) versus −24 g per week (95% confidence interval −36 to −12)) [26••].

A further complexity arises due to the difficult of achieving ‘blinding’ in brief intervention trials. Ideally, people who experience screening and brief alcohol interventions should be blinded to the alcohol focus of the study to reduce the possibility of social desirability bias and assessment reactivity in self-reported consumption levels at baseline and follow-up [37, 38]. Assessment reactivity (a type of research participant effect) is defined as:

the action of having a behavior queried, monitored, or become a focus of attention during a research study independently can affect the expression of that behavior regardless of other interventions or manipulations incorporated in the study [39]*.*

Nevertheless, some trials do achieve blinding by embedding alcohol into general health screening approaches and into advice or counselling about other health behaviours such as smoking. The use of digital platforms can make it easier to mask the alcohol focus on screening and brief interventions. However, it may be more difficult to ensure recipients actually receive the specific alcohol content as intended—and intervention fidelity can be hard to assess in practitioner-focused trials without intruding into the consultation, which could contaminate intervention effects. Once again, assessing intervention fidelity might be less problematic in digital trials where the material viewed can be tracked remotely. However, this raises ethical issues regarding surveillance.

One of the problems with synthesising evidence in the brief alcohol intervention field is the huge range of different outcomes reported. Some measures can be converted into a standard measure (such as grams of alcohol consumed per week), but many cannot. Systematic reviewers have a choice of omitting a proportion of trials from meta-analyses to facilitate a coherent pooled estimate, or using standardised mean difference, which can be difficult to convert into an intuitively meaningful outcome. Even alcohol consumption measures do not always measure the same thing—examples include the amount someone drinks over a week (quantity), the number of days they drink (frequency), the number of drinks in a single occasion (intensity) and the number of times they engage in a binge drinking session. When alcohol harms and health effects are reported, variability increases—several scales measure problems or negative consequences of drinking (e.g. [40]). There is some evidence that brief alcohol intervention may have a small but non-significant effect on presentation at the emergency department [41]. However, a narrative synthesis found evidence for the impact of days of hospitalisation as well as morbidity and mortality was inconclusive [42]. Secondary consequences, such as involvement in antisocial behaviour or crime, difficulties in interpersonal relationships and interpersonal violence, are less widely considered and show similar heterogeneity in form of measurement. Further research, and where possible evidence synthesis, is needed to establish the full impact (beyond consumption) of brief interventions in primary care. The ORBIT project, which has consulted practitioners and triallists about the most appropriate outcomes to report in brief intervention research, will provide useful direction for future work [43].

## Interpretation

Although most systematic reviews of brief alcohol intervention in primary care report a small, statistically significant decrease in alcohol consumption, debate has occurred about whether this effect is clinically meaningful. For example, if a person drinks more than 30 standard drinks per week, then how much difference does this reduction make? One argument, addressing the individual perspective, is that a focus solely on the change in consumption is incomplete because brief interventions also aim to change a person’s attitude to drinking and their relationship with alcohol. Brief interventions may provide a valuable shift part way along a behaviour change pathway that culminates in a reduction in consumption. A public health-focused argument would also suggest that even a small reduction in consumption across the large (and growing) population that drinks excessively would have a large effect at a population level on incidence of disease and harms.

It is important not to over-interpret trials that report null effects using null hypothesis significance testing [44]. A trial reporting a null effect demonstrates absence of evidence for an effect of the intervention, but does not demonstrate evidence that the null hypothesis is true. Heather discusses several reasons why participants in the intervention group of a brief intervention trial may not display a significant reduction in alcohol consumption including regression to the mean, research participation effect, historical trends and ecological effects (for example introduction of drink-driving laws or changes in recommended limits) or assessment reactivity [44].

Some trials report no significant difference between intervention and control participants, but this often belies the fact that participants in all arms reduced their consumption. Consequently, when significant effects of brief alcohol intervention are found, these are typically over and above reductions also occurring in control groups. Further investigation is needed to shed light on what is causing participants across all arms to cut down, and what extra brief interventions deliver compared to ‘control group’ conditions. In trials where no significant between-group effect is identified, it is important to assess changes over time and investigate whether active ingredients (e.g. screening) act across all arms, rather than dismissing intervention effects.

A final debate concerns whether brief intervention effects can be achieved in the ‘real world’, when they are administered to wider groups of patients and the content and fidelity of the intervention may differ. Pragmatic trials are best to assess real-world effectiveness. Here, the population and exact intervention are not tightly controlled, and researchers have little input into recruitment, delivery and follow-up. Efficacy trials are the opposite extreme or aim to maximise internal validity and determine whether the interventions can work in ideal (or the best possible) conditions. Some have argued that all brief intervention trials are effectiveness trials because brief interventions are designed for generalist practitioners to deliver to unselected populations [45]. However, many trials had significant researcher input that would not be available in primary care. Earlier work to assess the impact on the outcome of these trial design issues was inconclusive [26••]. More recently, three validated scales have emerged to guide more detailed assessment of whether trials are efficacy or effectiveness trials. RITES was specifically designed to assess trials retrospectively for systematic reviews [46], PRECIS-2 is a tool for the prospective design of trials [47, 48], and the earliest tool for differentiating trials was published in 2006 [49]. Work is underway to understand how the issue of efficacy or effectiveness affects heterogeneity in meta-analyses more generally [50], and this should help inform future assessment related to brief alcohol intervention. However, much of this debate is not relevant to alcohol interventions delivered via digital technology, which also finds positive effects of alcohol intervention. Here, the key limitation in evaluation terms is the relatively high rates of attrition or loss of participants at follow-up.

## Conclusions

Systematic reviews and meta-analyses have identified a significant effect of both practitioner-delivered and digital interventions, resulting in reductions in alcohol consumption of 20 g (practitioner-delivered) and 23 g (digitally-delivered) per week. Although this effect is small, it has the potential to impact at both individual and population levels. Future research should continue to focus on identifying the specific active components of these interventions and the contextual factors that optimise effects. Newer methods such as network meta-analysis could be employed to allow for both direct and indirect comparisons between trials. This will facilitate the refining of both content and delivery in order to maximise effectiveness. It is important that such investigations consider not only trials where a significant intervention effect is found, but also those which identify changes over time in multiple trial arms.

Whilst the evidence for the effect of interventions on alcohol consumption is well established, the effect on alcohol-related harms and secondary consequences requires further research attention. Consensus on the most appropriate outcomes to assess and measurement methods will facilitate future synthesis of evidence in this area allowing a full picture of the effectiveness of these interventions to emerge.

Finally, issues remain with regard to the ability of brief alcohol interventions to reach the target population on hazardous and harmful drinkers. Many studies have pointed to barriers to implementation of brief intervention in healthcare settings, such as availability of monetary resources, clinician time and adequately trained members of staff [51]. By bypassing the need for clinician involvement, digital interventions may be able to overcome some of the barriers to uptake among harmful and hazardous drinkers. Specifically, digital interventions are not subject to restrictions of geographical distance, can be accessed at a time and place of the individual’s choice and afford the drinker a greater sense of anonymity.
